# Association of some dietary ingredients, vitamin D, estrogen, and obesity polymorphic receptor genes with bone mineral density in a sample of obese Egyptian women

**DOI:** 10.1186/s43141-021-00127-0

**Published:** 2021-02-09

**Authors:** Nayera E. Hassan, Salwa M. El Shebini, Sahar A. El-Masry, Nihad H. Ahmed, Ghada Nour Eldeen, Enas A. Rasheed, Manal M. Aly, Khhadija M. Alian, Mahmoud A S. Afify, Aya Khalil

**Affiliations:** 1 Biological Anthropology Department, Medical Research Division, National Research Centre, Giza, Egypt; 2 Nutrition and Food ScienceDepartment, National Research Centre, Giza, Egypt; 3 Molecular Genetics and Enzymology Department, National Research Centre, Giza, Egypt; 4 Clinical and Chemical Pathology Department, Medical Research Division, National Research Centre, Giza, Egypt

**Keywords:** Osteoporosis, Vitamin D receptors genes, Estrogens receptors genes, Obesity receptors genes, Dietary intake

## Abstract

**Background:**

Although many environmental factors play an important role in bone mass density (BMD) variation, genetic influences account for 60–85% of individual variance. The aim of this study was to find the interaction between some dietary ingredients, vitamin D, estrogen, and obesity polymorphic receptor genes, among a sample of obese Egyptian women. This was a cross sectional study included 97 women (aged 25–60 years). Data on anthropometry, dietary intake, BMD, biochemical, and genetic analyses were collected.

**Results:**

Osteoporosis was high among women had dominant Taq1 vitamin D receptor gene while osteoporosis was less common among the homozygous Apa1 receptor gene women. Both genes in their two forms did not show any effect on serum vitamin D. Heterozygous types of osteoporotic women carried both genes revealed a slight but significant decrease in level of serum calcium. Xba1 estrogen receptor gene was identified only in a homozygous type while the heterozygous Pvu11 estrogen receptors gene has been identified among both osteoporotic and non-osteoporotic women, this gene was associated with higher BMI in both groups compared to the homozygous receptor gene. Mutant types of genotype FTOrs99 and FTOrs80 obesity receptors genes were less common (4.44%, 11%) among participants. Both of these genes were associated with the highest value of BMI and caloric daily intake, fat, and saturated fatty acid that were more prominent among osteoporotic women.

**Conclusion:**

There is significant association between vitamin D, estrogen, obesity receptors genes, special nutrients, and osteoporosis. Increased BMI, calories, and fat intake lead to rise of genetic predisposition and susceptibility to osteoporosis.

Background

The osteoporosis etiology is considered to be multifactorial related to polygenic background which can be modulated by integrated effects of genetic, hormonal, environmental and nutritional factors. Despite the fact that several environmental factors present an important role in the variation of bone mass density (BMD), the influence of genetic factors account for 60–85% of individual variance. However, genetic studies show that candidate genes are included in the BMD variation and also in the progression of osteoporosis [1, 2].

Vitamin D deficiency may be related to several factors as the exposure to sunlight and nutrition deficiency, in addition to race, age, sex, obesity, and impaired synthesis of vitamin D and its metabolism [3]. Vitamin D is obtained from same limited dietary sources and is derived from cutaneous synthesis upon sunlight exposure [4]. Throughout a multistep process that is highly regulated, vitamin D is metabolized to 1, 25-dihydroxyvitamin D which is a key hormone for regulating calcium homeostasis [4]. The hormone 1,25 dihydroxy vitamin D helps to regulate the gene expression that happens after being bonded to vitamin D receptor (VDR). It is a classic nuclear hormone receptor which is commonly expressed through all the body tissues [5, 6].

However, estrogen is known to be the key regulator hormone for bone metabolism in both women and men. Menopause which is accompanied by the loss of ovarian estrogens is associated with the decline in bone mineral density (BMD) [7]. The polymorphism that occurs to estrogen receptor genes (ESR1, ESR2) appears to be important among genetic factors [8]. Consequently, the gene encoding estrogen receptor 1; which is one of the two mediators of the action of estrogen; is considered to be an essential candidate in order to determine the risk of osteoporosis [9, 10]. Moreover, the associations of BMD and estrogen receptor polymorphisms in addition to lipids are incompatible [11].

Controversial findings have shown in different studies, regarding obesity impact on the bone metabolism. Salamat and his colleagues [12] revealed that obesity, that is defined by BMI, confirmed low BMD and decreased osteoporosis risk in a non-institutionalized population. Both lean mass and body fat were suggested to assist in BMD maintenance which occurs by generating mechanical overload on the bones [13, 14]. However, it was evident recently that sarcopenic obesity, which is a term that refers to the presence of increased body fat and decreased muscle mass, was reported to be accompanied with osteoporosis development among elderly population [15, 16]. The genome-wide associations studies (GWASs) have successfully identified loci which is associated with obesity and osteoporosis. There are also seven potentially pleiotropic genes had been identified to be associated with obesity and osteoporosis. The result of research’s and findings can provide recent insight into potential genetic determination and the codetermination mechanism of obesity and osteoporosis [17].

The objective of this study was to find the interaction between some dietary ingredients; and vitamin D, estrogen, and obesity polymorphic receptor genes, among a sample of obese Egyptian women.

## Methods

This cross-sectional study included 97 Egyptian obese women (with age range 25–60 years, and mean age 48.85 + 9.88 years.). They were recruited and randomly chosen, from all employees and workers of all categories of the “Blinded for peer review”. They were classified according to their BMD-t score into 2 groups: osteoporotic (*n* = 52) and non-osteoporotic (*n* = 45). A written informed consent was obtained from all participants after being informed about the purpose of the study. This research paper was derived from a cross-sectional survey of a project funded by Blinded for peer review, 2016–2019 entitled “Bone mass among Overweight and Obese Women: Mechanism and Intervention.” (Blinded for peer review), with an approval obtained from Ethics Committee of Blinded for peer review (Registration Number is 16/127).

The following parameters, anthropometric measurements, DEXA, laboratory and genetic investigations, and dietary intake were assessed for all participants.

### Anthropometric measurements

Body weight and height were measured, following the recommendations of the “International Biological Program” [18]. Body weight (Wt) was determined to the nearest 0.01 kg using a Seca Scale Balance, with the participant wearing minimal clothes and with no shoes. Body height (Ht) was measured to the nearest 0.1 cm using a Holtain portable anthropometer. Body mass index [BMI: weight (in kilograms) divided by height (in meters squared)], was calculated.

### DEXA measurements

Bone mineral density “BMD” (gm/cm^2^) at the neck of femur were measured using dual-energy DEXA (DEXA Norland XR-46 version 3.9.6/2.3.1, USA). Full body DEXA scan, based on the woman’s age, weight, and height, was performed with the participant keeping the precise distance between her arms and legs according to the machine instructions manual. A well-qualified operator executed and evaluated all analyses using the same protocol for all assessments. The instruments were calibrated daily according to the manufacturer’s instructions. Osteoporosis is established by measurement of BMD at the neck of femur using the *T* score which was calculated using the following formula: *T* score = (measured bone density-maximum bone density)/the maximum standard deviation;

if *T* score ≥ − 1.0 was grouped as normal, *T* score < − 1.0 to > − 2.5 was put in the osteopenia and *T* score ≤ − 2.5 were categorized as having osteoporosis following the diagnostic criteria established by the World Health Organization [19] in adults. All participated women were divided into two groups: normal and osteopenia (45 non-osteoporotic) and 52 osteoporosis according to their bone health status.

### Laboratory investigations

After overnight 8 h fasting, participants’ venous blood samples were obtained by venipuncture in the morning to assess the following parameters: serum calcium (Ca) and serum 25 hydroxy vitamin-D. The blood samples that were left to clot were then centrifuged at 5000 rpm for 10 min to separate sera; that were then stored at – 80 °C to be assayed later on. The assessments of these parameters were done in the laboratory of “Medical Excellence Research Center MERC”, which is part of “Blinded for peer review”.

Serum calcium level was measured using the automated clinical chemistry analyzer Olympus AU 400 analyzer [20]. Serum 25 hydroxy vitamin-D (25 (OH) D) was assessed by ELISA kit, for vitamin D Catalogue number SL1831 HU.Sun long Biotech.Co. Ltd. [21].

### Genetic investigations

#### Extraction of genetic material and polymorphisms analysis

**Fig. 1:**
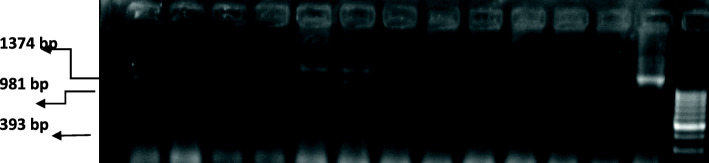
Detection of Xba1 polymorphism. PCR and digestion products. Lanes 1, 2, and 6: X/x genotype (1374, 981, and 393 bp). Lanes 3, 4, 5, 7, 8, 9, 10, 11, and 12: X/X genotype (1374 bp). Lane 13: the PCR amplified product (1374 bp). M: ΦX 174 marker/Hae III digest

DNA was extracted from the blood samples by using QIAamp DNA mini kit (QIAGEN, Germany). The presence of PvuII and XbaI polymorphisms (Fig. 1) within the ESR gene were analyzed using polymerase chain reaction-restriction fragment length polymorphism (PCR-RFLP). The oligonucleotide primers used to determine the PvuII and XbaI polymorphisms included forward primer, 5′-CTG CCA CCC TAT CTG TAT CTT TTC CTA TTC TCC- 3′; and reverse primer, 5′-TCT TTC TCT GCC ACC CTG GCG TCG ATT ATC TGA- 3′. PCR reactions were performed through 30 cycles by the following: 50 s at 95 °C (denaturation), 50 s at 62 °C (annealing), 50 s at 72 °C (extension), and final extension for 7 min at 72 °C to ensure a complete extension of all PCR products.

**Fig. 2:**
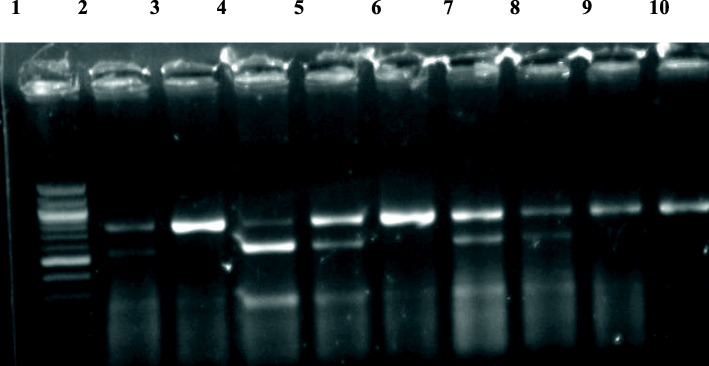
Gel image of VDR gene ApaI polymorphism PCR-RFLP products. Lane 1: Molecular size marker (100 bp, Vivantis). Lanes 3, 6, 9, 10: **AA** wild-type homozygosis fragment 740 bp. Lane 4: **aa** mutant homozygosis fragments of 530 and 210 bp. Lanes 2, 5, 7, 8: **Aa** heterozygosis produces fragment of 740, 530, and 210 bp

**Fig. 3:**
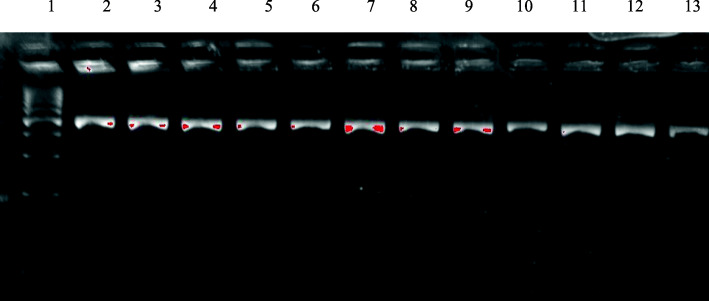
Gel image of VDR gene TaqI polymorphism PCR-RFLP products. Lane 1: Molecular size marker (100 bp, Vivantis). lanes 2, 5, 6, 10, 11,13: **TT** wild- type homozygosis produces fragments of 495 bp. lanes 3, 4, 7, 8, 9,12: **Tt** heterozygosis produces fragments of 495, 290, and 205 bp

All the included cases were genotyped using PCR-RFLP for two restriction sites in the VDR gene; ApaI (rs7975232) (A/C) in intron 8 (Fig. 2) and TaqI (rs731236) (A/G) in exon 9 (Fig. 3) using specific primer sequences; these primers were forward: 5′GGGATGGACAGAGCATGG3′ and reverse: 5′CCACCTCCCCTATCCACC3. Genomic DNA was amplified using the following: initial denaturation at 94 °C for 10 min, and 30 cycles of denaturation at 94 °C for 1 min, annealing at 64 °C for 1 min and extension at 72 °C for 1 min, followed by final extension at 72 °C for 10 min.

### Post-PCR-RFLP

The resulting DNA fragments were subjected to restriction digestion using the respective enzymes. The genotypes were resolved on 3% (w/v) agarose gels. Genotypes were designated conventionally by the first letter of the name of the enzyme. Capital letter indicates the absence of the cut site, whereas lower case letter indicates its presence.

The FTO rs9939609 and rs8050136 SNPs were genotyped by the Taqman assay (ABI: Applied Biosystems, Foster City, USA). The primers and probes of SNPs were from ABI assay on demand kit. Reactions were carried out according to the manufacturer’s protocol. The probe fluorescence signal detection was performed using the ABI Prism 7900 real-time PCR system.

### Dietary intake

Detailed data about nutritional habits and intake through: 24 recall diet history was collected. Analysis of food items, particularly calcium, vitamin D, and fat (saturated, mono, and poly unsaturated fat) intake, was done using World Food Dietary Assessment System, (WFDAS) [22], USA, University of California.

### Statistical analysis

Data were analyzed using the Statistical Package for Social Sciences (SPSS/Windows Version 22, SPSS Inc., Chicago, IL, USA). Normality of data was tested using the Kolmogorov-Smirnov test. Some of the variables, such as the data of DEXA, weight, BMI, and calcium intake were not normally distributed.

The parametric data were expressed as mean + SD, where the qualitative ones were expressed as number and percentage (%). The distribution of the women and the type of gene for the 2 groups were analyzed and compared using chi-square test for independent groups. Student’s *t* test was used to compare between 2 parametric groups. While ANOVA test was used to compare between 3 parametric groups. *P* value < 0.05 was regarded as statistically significant for all tests.

## Results

Distribution of women by the type of vitamin D, estrogen, and of obesity polymorph receptor genes are showed in Table 1. Osteoporosis was high among women had dominant Taq1 vitamin D receptor gene (65.38%), while the homozygousApa1 receptor gene might give a sort of protection against osteoporosis (80.0% of non-osteoporotic). No mutant Xba1 and Pvu11 estrogen receptor genes were detected among the studied sample, while the homozygous Xba1 was identified among all the participants. Distribution of the homozygous Pvu11 was higher among non-osteoporotic women while the heterozygousPvu11 have been identified at higher level among osteoporotic women. Both the genotype FTOrs99 and FTOrs80 obesity receptor genes were detected among non-osteoporotic and osteoporotic women with slight insignificant difference. The mutant types were less common, 4.44% and 11.54% among non-osteoporotic and osteoporotic women respectively. However, insignificant differences between the two non-osteoporotic and osteoporotic groups regarding the distribution of different genotypes were found.

**Table 1:** Distribution of genotypes of vitamin D receptors, estrogen receptors, and obesity receptors in studied non-osteoporotic and osteoporotic women

Parameters	Non-osteoporotic ***N*** = 45 %46.39	Osteoporotic ***N*** = 52 %53.61	Chi-square ***P***
**Taq1 vit. D receptor gene**			
**Homozygous**	26 (57.78%)	34 (65.38%)	0.442
**Heterozygous**	19 (42.22%)	18 (34.62%)	
**Mutant**	0.0 (0.0%)	0.0(0.0%)	
**Apa1 vit. D receptor gene**			
**Homozygous**	36(80.0%)	33 (63.46%)	0.162
**Heterozygous**	9 (20.0%)	18 (34.62%)	
Mutant	0 (0.00%)	1(1.92%)	
**Xba1 estrogen receptor gene**			
**Homozygous**	45 (100%)	52 (100%0)	-------
**Heterozygous**	0.0	0.0	
**Mutant**	0.0	0.0	
**Pvu11 estrogen receptor gene**			
**Homozygous**	32 (71.11%)	35 (67.31%)	0.686
**Heterozygous**	13(28.89%)	17 (32.69%)	
**Mutant**	0.0	0.0	
**FTOrs99 obesity receptor gene**			
**Homozygous**	27 (60.00%)	30 (57.69%)	0.615
**Heterozygous**	16 (35.56%)	17 (32.69%)	
**Mutant**	2 (4.44%)	5 (11.11%)	
**FTOrs80 obesity receptor gene**			
**Homozygous**	27 (60.00%)	24 (46.15%)	0.268
**Heterozygous**	16 (35.56%)	22 (42.31%)	
**Mutant**	2 (4.44%)	6 (11.54%)	

Comparisons between different studied vitamin D (Table 2), estrogen (Table 3), and obesity receptor^’^s genes (Table 4) genotypes regarding the related investigated characteristics parameters of the non-osteoporotic and osteoporotic were done. Both Taq1 and APal vitamin D receptor genes in their two forms did not show any effect on the vit. D and calcium intake, and serum vit. D. While heterozygous types of the osteoporotic women carried both Taq1 and APal vitamin D receptor genes revealed significant slight decrease in the level of serum calcium (Table 2). The Xba1 estrogen receptor gene was identified only in a homozygous type. The heterozygoustypePvu11 estrogen receptor gene was associated with insignificant higher BMI in both non-osteoporotic and osteoporotic women compared to the homozygous (Table 3). Among the osteoporotic women, both the mutant types of the FTOrs 99 and 88 obesity receptor genes were associated with the significant highest value of BMI (Table 4). In addition, the mutant types of the FTOrs 99 obesity receptor genes were associated with the significant highest value of caloric intake, fat intake, and saturated fatty acid intake (SFAs). Moreover, among the non-osteoporotic women, both the heterozygous types of the FTOrs 99 and 88 obesity receptor genes were associated with the significant highest value of caloric intake and insignificant highest value of BMI, while the mutant types were associated with the significant highest value of saturated (SFAs) and mono-unsaturated fatty acid intake (MUFAs).

**Table 2:** Comparison between different studied vitamin D receptors genes genotypes regarding the related investigated parameters

Parameters ***N*** = 97	Non-osteoporotic ***N*** = 45			Osteoporotic ***N*** = 52		
**Taq1 vit. D receptor**	**Homozygous** **No: 26**	**Heterozygous** **No: 19**	***P***	**Homozygous** **No: 34**	**Heterozygous** **No: 18**	***P***
	**Mean ± SD**	**Mean ± SD**		**Mean ± SD**	**Mean ± SD**	
**Age (years)**	38.39 **±** 7.11	38.68 **±** 9.05	0.907	54.96 **±** 5.15	53.41 **±** 5.61^* #^	0.322
**Weight (kg)**	79.34 **±** 20.21	78.64 **±** 22.35	0.885	81.52 **±** 12.80	72.75 **±** 21.37	0.135
**Height (cm)**	157.69 **±** 4.42	155.95 **±** 6.95	0.229	155.62 **±** 4.77	152.00 **±** 9.09^* #^	0.156
**BMI kg/m**^**2**^**)**	31.89 **±** 7.91	31.96 **±** 9.93	0.978	33.78 **±** 5.85	31.77 **±** 1.07	0.386
**Vit D intake( ųg)**	7.22 **±** 1.80	6.59 **±** 2.71	0.622	5.81 **±** 2.04	6.88 **±** .3.24	0.976
**Serum vit. D (ng/mL)**	25.53 **±** 8.60	23.11 **±** 6.56	0.389	23.18 **±** 9.25	25.44 **±** 6.45	0.410
**Calcium intake( mg)**	689.13 **±** 25.80	613.54 **±** 27.24	0.164	604.17 **±** 28.58.	601.70 **±** 29.65^* #^	0.515
**Serum calcium (mg/dL)**	9.92 **±** 0.81	9.28 **±** 0.67	0.081	9.26 **±** .0.77	9.19 **±** .0.59	0.014*
**Apa1 vit. D receptor**	**Homozygous** **No: 36**	**Heterozygous** **No: 9**	***P***	**Homozygous** **No: 33**	**Heterozygous** **No: 18**	***P***
	**Mean ± SD**	**Mean ± SD**		**Mean ± SD**	**Mean ± SD**	
**Age (years)**	38.39 **±** 7.76	39.24 **±** 9.24	0.317	55.03 **±** 4.48	53.48 **±** 6.62	0.383
**Weight (kg)**	75.35 **±** 2.04	93.81 **±** 3.29	0.036*	80.48 **±** 1.61	73.86 **±** 1.70	0.131
**Height (cm)**	156.57 **±** 5.26	156.96 **±** 5.62	0.245	153.94 **±** 6.75	154.89 **±** 6.94^* #^	0.814
**BMI Kg/m**^**2**^**)**	30.67 **±** 7.94	31.92 **±** 8.71	0.056	34.28 **±** 8.44	30.62 **±** 6.28	0.107
**Vit D intake( ųg)**	6.20 **±** 1.47	5.91 **±** 2.33	0.632	6.16 **±** 2.48	6.13 **±** 1.72	0.621
**Serum vit. D (ng/mL)**	24.80 **±** 8.16	24.54 **±** 7.84	0.513	25.78 **±** 9.83	21.11 **±** 3.62	0.077
**Calcium intake( mg)**	686.53 **±** 25.19	618.77 **±** 27.63	0.474	609.19 **±** 24.35	607.49 **±** 29.31	0.172
**Serum calcium (mg/dL**	9.61 **±** 0. .60	9.32 **±** 0.62	0.332	9.14 **±** 0.52	9.08 **±** 0.59	0.010*

*BMI* body mass index, *vit. D* Vitamin D

*P < 0.05 homozygous vs. heterozygous

#*P* < 0.05 non-osteoporotic vs. osteoporotic

**Table 3:** Comparison between different studied estrogen receptors genes genotypes regarding the related investigated parameters

Parameters ***N*** = 97	Non-osteoporotic ***N*** = 45			Osteoporotic ***N*** = 52		
**Taq1 vit. D receptor**	**Homozygous** **No: 26**	**Heterozygous** **No: 19**	***P***	**Homozygous** **No: 34**	**Heterozygous** **No: 18**	***P***
	**Mean ± SD**	**Mean ± SD**		**Mean ± SD**	**Mean ± SD**	
**Xba1 estrogen receptors**	**Homozygous** **No: 45**	**Heterozygous** **No: 0.0**		**Homozygous** **No: 52**	**Heterozygous** **No: 0.0**	
	**Mean ± SD**	**Mean ± SD**		**Mean ± SD**	**Mean ± SD**	
**Age (years)**	38.55 **±** 7.97	38.68 **±** 9.05	0.907	54.39 **±** 5.30^*^	53.41 **±** 5.61^*^	0.322
**Weight (kg)**	79.04 **±** 2.41	78.64 **±** 22.35	0.885	78.48 **±** 1.66	72.75 **±** 21.37	0.135
**Height (cm)**	156.96 **±** 5.62	155.95 **±** 6.95	0.229	154.37 **±** 6.73^*^	152.00 **±** 9.09^*^	0.156
**BMI (kg/m**^**2)**^	31.92 **±** 8.71	31.96 **±** 9.93	0.978	33.08 **±** 7.83	31.77 **±** 1.07	0.386
**Pvu11 estrogen receptor**	**Homozygous** **No: 32**	**Heterozygous** **No: 13**	***P***	**Homozygous** **No: 35**	**Heterozygous** **No: 17**	***P***
	**Mean ± S D**	**Mean ± SD**		**Mean ± SD**	**Mean ± SD**	
**Age (years)**	38.20 **±** 7.13	39.40 **±** 1.01	**0.638**	54.19 **±** 6.11	54.81 **±** 3.18^* #^	0.657
**Weight (kg)**	75.94 **±** 2.07	86.68 **±** 3.07	0.193	77.58 **±** 1.50	80.35 **±** 1.98	0.538
**Height (cm)**	156.62 **±** 4.36	157.77 **±** 8.11	0.649	154.37 **±** 5.52	154.35 **±** 8.92^* #^	0.904
**BMI (kg/m**^**2**^**)**	30.86 **±** 7.96	34.53 **±** 1.02	0.205	32.53 **±** 6.23	34.21 **±** 1.05	0.474

*BMI* body mass index

#*P*, 0.05 non-osteoporotic vs. osteoporotic

**Table 4:** Comparison between different studied obesity receptors genes genotypes regarding the related investigated parameters

Parameters ***N*** = 97	Non-osteoporotics ***N*** = 45				Osteoporotic ***N*** = 52			
**FTOrs99 obesity receptor**	**Homozygous** **No: 27**	**Heterozygous** **No: 16**	**Mutant** **No: 2**	***P***	**Homozygous** **No: 30**	**Heterozygous** **No: 17**	**Mutant** **No: 5**	***P***
	**Mean ± SD**	**Mean ± SD**	**Mean ± SD**		**Mean ± SD**	**Mean ± SD**	**Mean ± SD**	
**Age (years)**	36.65 **±** 8.01#	41.75 **±** 7.17	38.63 **±** 9.75	0.134	55.20 **±** 4.68#	51.93 **±** 5.91┼	57.92 **±** 3.77	0.040*
**Weight (kg)**	74.12 **±** 2.71	87.61 **±** 1.53	77.00 **±** 3.47	0.183	79.82 **±** 1.57	74.11 **±** 1.79	85.32 **±** 1.63	0.283
**Height (cm)**	157.30 **±** 6.01	157.19 **±** 4.32	150.50 **±** 9.19	0.234	155.17 **±** 5.52	155.18 **±** 6.85┼	146.80 **±** 9.44@	0.027*
**BMI (kg/m**^**2)**^	29.77 **±** 9.67#	35.38 **±** 5.54	33.24 **±** 3.12	0.120	33.18 **±** 6.44	30.62 **±** 6.88┼	40.83 **±** 5.38@	0.034*
**Caloric intake (Cal)**	1655.93 **±** 28.75#	2483.73 **±** 26.19	2150.64 **±** 8.76	0.007**	2684.63 **±** 37.69#	2711.63 **±** 31.15	2720.13 **±** 39.54	0.030*
**Fat intake (g)**	85.71 **±** 2.97	90.55 **±** 8.26	114.18 **±** 13.61	0.554	132.38 **± 1**5.54#	134.25 **±** 13.34	135.14 **±** 14.50	0.017*
**SFAs (g)**	25.57 **±** 16.01#	32.88 **±** 12.95	37.21 **±** 8.70	0.002**	44.67 **±** 17.21#	46.14 **±** 15.82	47.30 **±** 15.46	0.014*
**MUSFAs (g)**	18.73 **±** 16.02#	20.34 **±** 13.78	20.96 **±** 2.78	0.005**	32.75 **±** 16.12#	31.72 **±** 20.04	31.36 **±** 14.69	0.114
**PUSFAs (g)**	18.83 **±** 18.45#	21.85 **±** 13.41	20.44 **±** 14.74	0.072	30.23 **±** 18.48	31.64 **±** 8.95	29.63 **±** 15.04	0.329
**FTOrs80 obesity receptor**	**Homozygous** **No: 27**	**Heterozygous** **No: 16**	**Mutant** **No: 2**	***P***	**Homozygous** **No: 24**	**Heterozygous** **No: 22**	**Mutant** **No: 6**	***P***
	**Mean ± SD**	**Mean ± SD**	**Mean ± SD**		**Mean ± SD**	**Mean ± SD**	**Mean ± SD**	
**Age (years)**	36.65 **±** 8.015#	41.75 **± 7.17#**	38.63 **±** 9.75	0.134	55.28 **±** 4.86	52.48 **±** 5.62┼	57.84 **±** 3.38^*^	0.051
**Weight (kg)**	74.12 **±** 2.71	87.61 **±** 1.53	77.00 **±** 3.47	0183	77.15 **±** 1.57	77.58 **±** 1.78	87.13 **±** 1.52	0.389
**Height (cm)**	157.30 **±** 6.01	157.19 **±** 4.32	150.50 **±** 9.19	0.234	154.46 **±** 5.47	156.09 **±** 6.57┼	147.67 **±** 8.71@	0.027*
**BMI (k/m**^**2)**^	29.77 **±** 9.67#	35.38 **±** 5.54#	33.24 **±** 2.12	0.120	32.40 **±** 6.66	31.67 **±** 6.60┼	40.96 **±** 4.23^@^	0.027*
**Caloric intake (Cal)**	1655.93 **±** 28.75#	2483.76 **±** 26.19#	2450.65 **±** 28.76	0.007**	2265.28 **±** 29.75	2625.97 **±** 28.69	2711.85 **±** 26.91^*^	0.314
**Fat intake (g)**	90.55 **±** 8.26	114.18 **±** 3.61	118.71 **±** 2.97	0.554	120.43 **±** 4.86	129.79 **±** 5.97	131.90 **±** 3.31	0.137
**SFAs (g)**	20.57 **±** 16.01#	36.88 **±** 12.95#	38.21 **±** 8.70	0.002**	36.14 **±** 16.98	44.46 **±** 18.47	46.76 **±** 14.23^*^	0.182
**MUSFAs (g)**	18.73 **±** 6.02#	25.34 **±** 13.78#	25.96 **±** 10.78	0.005**	26.84 **±** 11.02	30.12 **±** 11.13	29.69 **±** 10.70^*^	0.481
**PUSFAs (g)**	18.83 **±** 4.45#	21.85 **±** 10.#41	22.44 **±** 11.74	0.072	24.37 **±** 10.72	29.58 **±** 11.14	25.06 **±** 7.24^*^	0.491

*BMI* body mass index, *SFAs* saturated fatty acids, *MUSFAs* mono unsaturated fatty acids, *PUSFAs* poly unsaturated fatty acids

^#^Significant differences between homo and hetero

^┼^Significant differences between hetero and mutant

^@^Significant differences between mutant and homo

## Discussion

The role of nutrition on bone health is currently very important research area. Important function of vitamin D is to control calcium homeostasis through increasing intestinal absorption, as well as calcium bone restoration, and reduce parathyroid hormone (PTH) [23].

Osteoporosis; a multifactorial illness characterized by a decrease in bone mineral density which increments the likelihood of bone fractures; is caused by calcium insufficiency, and its rate increments with age. It is known that mutations in the functional regions of vitamin D receptor gene will influence the metabolism of minerals particularly calcium and so bone metabolism [24].

However, in this study, no effect for these genes was observed on the levels of serum vitamin D. Zakiet al [25]. stated that obese Egyptian women carry polymorphic alleles showed significant lower levels of serum 25(OH) D. As for the level of serum calcium, data of this study showed that the heterozygous types of the osteoporotic women carried both genes revealed a slight but significant decrease in the level of serum calcium when compared to the homozygous osteoporotic women. In this context, Rivera-Leon et al. [26] found that the TT genotype of TaqI VDR gene polymorphism was correlated with low levels of osteocalcin (OC) in overweight and obese subjects.

Estrogen receptor 1 has an important role in the maintenance of the skeletal system which has been proven in experimental mice, from which the gene was deleted from the specific bone cells and their precursors. Absence of the estrogen receptor in osteoblast progenitor and precursor cells influenced the periosteum, whereas their deficiency in differentiated osteoblasts, osteoclasts, and osteocytes come about in diminished cancellous bone mass [27]. The results of this study showed that the homozygous Xba1 receptors gene was identified in all the women either non-osteoporotic or osteoporotic which might indicate no direct relation to osteoporosis. In the evaluations on ERα gene XbaI polymorphism and COL1A1gene Sp1 polymorphism, it was reported that there was no distinction in terms of average BMD values, genotype, and allele frequencies among groups. However, Mondockova et al. [28] expressed that decreased BMD in postmenopausal women may be linked to the Xba1 polymorphism. The homozygous estrogen receptor gene Pvu11 was slightly more prevalent among non-osteoporotic women, while its polymorph heterozygous was found slightly more among osteoporotic participants (32.69% of the osteoporotic compared to 28.89% of the non-osteoporotic women). It was assigned in postmenopausal women that ERα gene PvuII polymorphism was effective on the BMD values of the lumbar vertebra [29].

It was already accepted that obesity and osteoporosis were two different diseases, but later researches have revealed that both diseases share many common genetic and environmental factors [30]. Genetic inclination to weight gain may have connecting with an obesogenic environment. Developing researchers have found that changes in adiposity and metabolic reaction to low-calorie weight diets may well be altered by hereditary variations related to weight gain, metabolic condition and inclination to some kinds of foods [31]. Current study revealed that both the mutant types of the FTOrs 99 and 88 obesity receptor genes were associated with the highest value of BMI and also higher intake of calories, fat and saturated fatty acid (SFAs), which were more prominent among the osteoporotic women. Franzago et al. (2020) reported that nutrition may be a modifiable key that is able to be associated with both the genome and epigenome to impact the health of the human being. In specific, dietary components, nutrient requirements, and the diet itself are able to modulate gene expression [32].

## Conclusion

Osteoporosis was high among women had dominant Taq1 vitamin D receptor gene, while it was less prevalent among women had homozygous Apa1 receptor gene. However, both genes had no effect on either vitamin D or calcium intake, or the serum level of vitamin D. Yet, the osteoporotic women carried heterozygous types of both genes revealed a slight but significant decrease in the level of serum calcium. This may be due to the lack of calcium absorption among them, which need to increase their awareness to increase their intake of calcium rich foods. The mutant genotypes FTOrs99 and FTOrs80 obesity receptor genes might predispose to significant genetic variation in the intake of calories and fat; especially the saturated fatty acids; plus the BMI status. Based on these results, these people should be instructed to avoid or decrease the consumption of such foods and in addition reduce their weight.

## Data Availability

The datasets used and/or analyzed during the current study are available from the corresponding author on reasonable request, after taking the permission of our institute “National Research Centre.”

## Abbreviations

BMD: Bone mass density
BMI: Body mass index
ESR1, ESR2: Estrogen receptor genes
Ht: Body height
GWASs: Genome-wide association’s studies
OC: Osteocalcin
SFAs: Saturated fatty acid
SPSS: Statistical Package for Social Sciences
WFDAS: World Food Dietary Assessment System
WT: Body weight
VDR: Vitamin D receptor (VDR)
(25 (OH) D): Serum 25 hydroxy vitamin-D
